# Big data in mental health research – do the *n*s justify the means? Using large data-sets of electronic health records for mental health research

**DOI:** 10.1192/pb.bp.116.055053

**Published:** 2017-06

**Authors:** Peter Schofield

**Affiliations:** 1 King's College London

## Abstract

Advances in information technology and data storage, so-called ‘big data’, have the potential to dramatically change the way we do research. We are presented with the possibility of whole-population data, collected over multiple time points and including detailed demographic information usually only available in expensive and labour-intensive surveys, but at a fraction of the cost and effort. Typically, accounts highlight the sheer volume of data available in terms of terabytes (10^12^) and petabytes (10^15^) of data while charting the exponential growth in computing power we can use to make sense of this. Presented with resources of such dizzying magnitude it is easy to lose sight of the potential limitations when the amount of data itself appears unlimited. In this short account I look at some recent advances in electronic health data that are relevant for mental health research while highlighting some of the potential pitfalls.

## Recent advances

The most extensive electronic health data available for research in the UK are collected in primary care. For example, the Clinical Practice Research Datalink (CPRD) covers approximately 5 million active patients, with longitudinal records going back to 1987. This in turn is now linked to hospital episode statistics (HES) and mortality data, providing one of the world's largest longitudinal health data-sets.^1^ As with any big data project much depends on the quality of the data. This may be enhanced in primary care, as general practitioners (GPs) have a financial incentive to accurately record certain treatments and outcomes under the quality and outcomes framework (QOF).^2^

While there is no national equivalent for psychiatric care, HES data provide at least some information about psychiatric in-patient stays nationally. There are also examples of local schemes providing comprehensive psychiatric data for research use, often on a very large scale. For example, the Case Register Interactive Search (CRIS) system covers the full clinical record of over 250 000 patients from the South London and Maudsley (SLAM) catchment area.^3,4^ This can be linked with neighbourhood census data, primary care records, HES data and educational data from the National Pupil Database (NPD). A feature of CRIS is that it comprises the entire clinical record so that much of what is available is in the form of free text which, through recent advances in the use of natural language processing (NLP) techniques, is now accessible for large-scale research.^4^ For example, a recent project used free-text-mining algorithms to extract information about cannabis use to investigate the relation with clinical outcomes for just over 2000 patients with first-episode psychosis.^5^ Another recent study supplemented coded diagnostic and treatment data with data extracted from free text to look at delays in treatment and diagnosis for patients presenting with bipolar disorder.^6^

With over 50 publications to date using this data-set, CRIS has proved particularly useful for research into mortality outcomes for people with severe mental illness,^7,8^ hard-to-reach groups such as homeless people^9,10^ and, more recently, services for people in the early stages of psychosis.^11,12^

These examples are, however, still limited to either specific geographical regions or a relatively small subsample of the population. We have, of course, recently come close to a universal data-set of health records with the, ultimately ill fated, care.data proposal. Originally intended to link primary care data with existing hospital records, this would have provided whole-population data for research use. Arguably, this was unsuccessful because it was presented in a way that failed to reassure the public their data would be safe.^13^ While this has now been scrapped, it is still the government's aim that something similar is implemented.^14^

Allowing whole-population health data to be made available for research has, however, long been an accepted part of life in Nordic countries. For example, since 1968 all Danish citizens have had a unique personal identification number allowing data linkage across a range of health, welfare, employment and education data.^15^ This arguably represents a gold standard for mental health research, with all psychiatric in-patient admissions (since 1969) and all out-patient contacts (since 1995) providing longitudinal data for the entire population over nearly five decades.^16^ Because of the scale of longitudinal data collected, register-based studies using data such as these have proved particularly useful for aetiological research into relatively rare disorders such as schizophrenia. For example, a number of landmark papers have highlighted urban/rural differences in psychosis incidence^17,18^ and also documented the increased risk of psychosis for migrants and refugees.^19,20^

## Do big data mean high-quality data?

All these developments in the resources available for research are to be welcomed. However, simply having the ability to access data on this scale is not enough. What we gain through the sheer volume of data and breadth of coverage could be offset by ill-informed analysis and interpretation that fails to account for the limitations of the data. One fundamental limitation is that almost all examples of what we think of as big data are collected for purposes other than research. Health records, just like any bureaucratic product, are shaped by administrative convenience rather than the search for scientific truth. For example, if we look at the way that depression is recorded in primary care, it would be a mistake to take this at face value.^21,22^ For some time, recording a diagnosis of depression on the electronic record has triggered a series of prompts and demands on the clinician, which many saw as unnecessarily burdensome. This became a disincentive to code a formal diagnosis and instead alternatives, such as ‘low mood’, would be entered, although treatment itself remained unaffected. This has meant that GP records can show an exceptionally low prevalence of depression compared with what we know from national survey data.^23,24^ In this case, a failure to understand what statisticians term the data-generating process would lead to a fundamental misinterpretation of what these data represent. Furthermore, the quantity of data collected here makes no difference to the validity of our conclusions. In fact, having more data is likely to help reinforce any erroneous claims.

Looking at health informatics more broadly, a classic example of what can go wrong if we fail to understand the data-generating process is that much cited example of big data, Google flu trends. Here, the frequency and location of a selection of Google search terms, based on health-seeking behaviour, were used to predict where and when the next flu epidemic would occur.^25^ This was shown to more accurately predict epidemics compared with previous epidemiological studies and was therefore held up as an exemplar of the ascendancy of big data in health research.^26^ That is, until Google flu trends stopped predicting accurately and eventually proved no better than estimates based on flu prevalence from a few weeks before.^27^ This was in part a result of changes Google had made to their search engine, including the introduction of the auto-complete feature that meant searches no longer worked in quite the same way as when the algorithm was first devised. This problem was further exacerbated as the original search terms were never actually made public so could not be externally validated. Clearly, electronic health records are not subject to the same technical issues as a search algorithm. However, as we outline above, changes in the data-generating process, such as how diseases are coded, could make an important difference to results. In some ways, Google flu trends is the perfect example of the hubris associated with big data; as one of the early evangelical accounts confidently stated, ‘society needs to shed some of its obsession with causality in exchange for simple correlations: not knowing why but only what’.^26^ Although this might make sense if we are simply mining data looking for patterns, this approach alone has little to offer in the way of research evidence.

## Are the data we routinely collect aligned with research agendas?

A further limitation of research using administrative data is that we rarely have any control over what is collected and therefore risk the research agenda being set by what data are available. One field in which there have been major advances in recent years is ethnicity and mental health, partly due to the availability of electronic health records where patients' ethnicity is now routinely coded. In particular, large-scale case registers have been used to document the increased incidence of psychosis among Black and minority ethnic groups, as well as exploring possible risk factors to explain these differences.^28–31^ These findings have been validated using other methodologies. However, there is a risk that we now focus research attention on what are often fairly crude categories, while neglecting other forms of minority status or more nuanced definitions of ethnicity simply because of the available data. For example, it is likely that other forms of marginalised status may also be relevant as risk factors where individual characteristics (such as sexuality, social class or marital status) are at variance with what is usual in a locality.^32,33^ However, these are typically not recorded in register data and are therefore unlikely to receive as much research attention. Where relevant risk factors are not being recorded, research has the potential to inform the data collection process to not only benefit research but also enhance clinical care.

## How complex is the analysis of big data?

Another inherent danger is in the way we analyse these data. Often, the more data we have to analyse the more likely it is that we miss patterns in the data that could confound the associations we are interested in. For example, there might be temporal patterns in longitudinal data, such as long-term disease trends, that make it difficult to distinguish effects in before-and-after study designs. Short-term events such as the shift from ICD-9 to ICD-10 in the 1990s could confound our results when comparing changes in rates of diagnosed psychiatric disorders. Data might also be spatially patterned, with different environmental risk factors operating in different areas. This might be further patterned by administrative structures where, for example, differences in mental health outcomes in particular areas may reflect the performance, and reporting practices, of different mental health trusts. Considerable advances have been made in recent years in the tools available for analysing data patterned in this way. In particular, multilevel modelling and Bayesian analysis techniques allow us to simultaneously account for effects operating at temporal, individual, spatial and administrative levels. However, these are still not easily accessible to many researchers, or research consumers, although their use and accessibility are increasing. Implicit in these methods is a fundamentally different approach to that of small-scale studies, such as randomised controlled trials, where the aim is to remove complexity from the data through random allocation. With big data we can no longer rely on random assignment and rely instead on being able to model the complexity inherent in the data to account for possible confounding effects.

## Do big data mean more or less transparency?

Admittedly, complex data of this kind can be difficult to analyse, but it also presents an ever-increasing number of choices about how the analysis could be conducted. We might use different diagnostic categories, we could follow our sample over different time periods and look at a variety of different subgroups. We might use different statistical methods for the same analysis and we could also adjust for different sets of covariates. This growing array of possibilities also increases the opportunities to pick and choose our analysis until we find the most impressive-looking *P*-value. This tendency, often termed *P*-hacking or *P*-fishing, can be found in any statistical analysis, unless of course the method is predetermined and published in an advance protocol. However, big data exacerbate this tendency by increasing the possibilities for analysis. Often this means that statistically significant effects, which appear to show something important, cannot then be reproduced and our analysis is ‘over-fitted’ to our data. The US statistician Andrew Gelman describes this potential as the ‘garden of forking paths’.^34^ He argues that this need not necessarily mean deliberate deception on the part of the analyst, but is often the result of unconscious bias as reasonable analysis decisions are made but they are contingent on the data. The accumulation of these decisions, at different stages in the analysis, ultimately leads to a statistically significant result being more likely. What is required, argues Gelman, is greater transparency so that we are able to retrace the steps made in the analysis to assess for ourselves the significance of findings. A related problem with large data-set analysis is that often very low, highly statistically significant *P*-values can be found for what amount to clinically insignificant effects. It is argued that these tendencies have led to what has been described as a ‘reproducibility crisis’ in science.^35^ In response, the American Statistical Association recently issued a statement calling for greater transparency in the reporting of results and a move away from simply reporting *P*-values below a certain threshold (*P*<0.05).^36^

## Complementary methods

Clearly, there are some inherent problems in the analysis of large-scale health records data, both for the unwary and for the unscrupulous. However, there is nothing either inherently good or bad about the use of these kinds of data for mental health research. Ultimately, this comes down to understanding the human story behind how the data were created, having the analytical skills to best interpret the data and being transparent in the way results are reported. What big data can then give us is one version of the truth to complement what we are able to discover using other methods. In fact, one of the best examples of big data that we have in UK mental health, CRIS, also includes a parallel community survey component, the South East London Community Health Study (SELCoH).^37^ This is intended both to provide a parallel sample of community controls to match the case register and to yield detailed information about individual circumstances and attitudes otherwise absent from medical records.

There are of course a number of well-established national community survey resources, such as the Adult Psychiatric Morbidity Survey and the annual Health Survey for England, that are not dependent on health service use or subject to the diagnostic bias that occurs in health records data.^38,39^ We must also not forget the potential for qualitative research to address many of the questions in mental health research that are beyond the reach of statistical analysis. With the increased emphasis on evidence-based medicine, qualitative methods have increasingly been sidelined. For example, the *BMJ* recently announced that, in future, qualitative studies would have a low priority in the journal.^40^ In response, 76 senior academics from 11 countries wrote an open letter calling for the journal to reconsider.^41^ They cite the complementary role that qualitative research can have, particularly where there is a failure to reproduce the results of analyses of large-scale health data-sets.

Last, let us not forget that the research we do is only meaningful in that it relates to the, essentially individual, experience of mental disorder. Whatever volume of data we analyse, whether we look at *n* = 100 or *n* = 1 000 000, ultimately we are interested in what this can tell us about the experience of *n* = 1.
